# Quantitative serology for SARS-CoV-2 using self-collected saliva and finger-stick blood

**DOI:** 10.1038/s41598-022-10484-6

**Published:** 2022-04-21

**Authors:** Christopher Campbell, Nikhil Padmanabhan, Daniel Romero, Jessica Joe, Mikias Gebremeskel, Navaratnam Manjula, Noah Wohlstadter, Rachel Wohlstadter, Paul Goodwin, Lillian Quintero, Jeff Debad, George Sigal, Jacob Wohlstadter

**Affiliations:** Meso Scale Diagnostics, LLC., 16020 Industrial Drive, Gaithersburg, MD 20877 USA

**Keywords:** Biomarkers, Antibodies

## Abstract

Convenient and widespread serology testing may alter the trajectory of the COVID-19 pandemic. This study seeks to leverage high-throughput, multiplexed serologic assays, which have been adopted as benchmarks for vaccine efficacy, to support large-scale surveys of SARS-CoV-2 immunity using finger-stick blood and/or saliva. Specifically, we optimized MSD’s serology assays, which were analytically validated for serum, to test self-collected finger-stick blood and saliva samples to identify prior infection. We show that these assays can be used with FDA-registered specimen collection devices to obtain quantitative measurements for self-collected samples. First, we show that salivary antibodies are stable without refrigeration or preservatives for at least 5 days. We selected classification thresholds for antibodies against SARS-CoV-2 N, RBD and Spike in finger-stick blood and saliva that provided 98% specificity in a set of individuals without known COVID-19 exposure. Using matched samples, we show that testing of saliva and finger-stick blood equivalently identified individuals with humoral responses to CoV-2 antigens. Moreover, we piloted a simple saliva collection kit that can be used to safely send samples through the mail using written instructions only. This work establishes key parameters to robustly assay self-collected finger-stick blood and saliva using quantitative immunoassays that could support large-scale serology testing.

## Introduction

COVID-19 is likely to remain a public health concern for many years. Serology assays that can provide population-wide monitoring of immunity and exposure to SARS-CoV-2 are going to be increasingly important as tools for monitoring disease prevalence and identifying outbreaks. Efficiency of large-scale serosurveillance would benefit from the use of a self-collected sample that can be easily mailed to clinical laboratories for testing, thus bypassing the need for sample collection at medical facilities or specialized testing sites and significantly reducing the logistical challenges of large-scale testing^1–4^. This approach also has the potential to significantly lower costs relative to systems based on home testing by taking advantage of the cost-efficiencies of high-throughput laboratories, while also providing more quantitative and accurate results.

One primary type of self-collected samples for serology is finger-stick blood. Devices for collection of finger-stick blood typically combine a spring-loaded lancet for piercing the finger and a membrane or swab onto which a controlled volume of blood from the finger can be absorbed^5^. The blood is allowed to dry for stability during transport and is then extracted in a diluent prior to analysis. Multiple SARS-CoV-2 serology tests using finger-stick blood are already available^5–8^.

Saliva and other oral fluids are attractive alternative sample types that can be easily self-collected without pain or the use of needles or lancets. Various devices and methods have been described for collecting oral fluids^9,10^. These include swabs or sponges for collecting saliva from under the tongue, or gingival crevicular fluid (a fluid secreted at the interface of the gums and teeth) from the surface of the gums. Swab-based methods offer convenience for some users, especially young children. They also reduce the need to handle or view liquid saliva, which some people find unpleasant. However, swabs require additional downstream handling to extract saliva from the swabs. A commercial device from Salvimetrics is available that enables a simple and clean protocol for collection of liquid saliva by drooling through a specially designed straw into a collection tube^10^. While saliva has been previously described as a useful self-collected sample for serology, and is the sample used in an FDA-cleared serology test for HIV^11,12^, saliva-based serology tests for SARS-CoV-2 are not yet commercially available. The potential of saliva as a sample for SARS-CoV-2 serosurveillance is supported by the strong correlation that has been observed in the antibody response in serum and saliva during acute infection and recovery^13–17^.

MSD recently developed quantitative, multiplexed serology assays for SARS-CoV-2 that have been widely used for monitoring immune responses to SARS-CoV-2 vaccines in clinical trials^18,19^, correlate with viral neutralization^20^, and are potential correlates of immunity^21,22^. In this report, we describe an integrated approach for applying these assays to measuring SARS-CoV-2 antibody responses in mailed, self-collected saliva that provides a scalable framework for large-scale serosurveillence studies. The work presented here addresses fundamental questions associated with the testing of self-collected and mailed specimens, and establishes a strong foundation for future clinical performance studies. We demonstrate that users can successfully collect and ship samples, shipping can be achieved in adherence with regulations for shipping clinical diagnostic specimens, and samples are stable under typical shipping conditions. In addition, we establish normal ranges for saliva samples, and demonstrate a correlation between antibody levels in saliva and finger-stick blood.

## Results

### Stability of salivary antibodies

**Figure 1:**
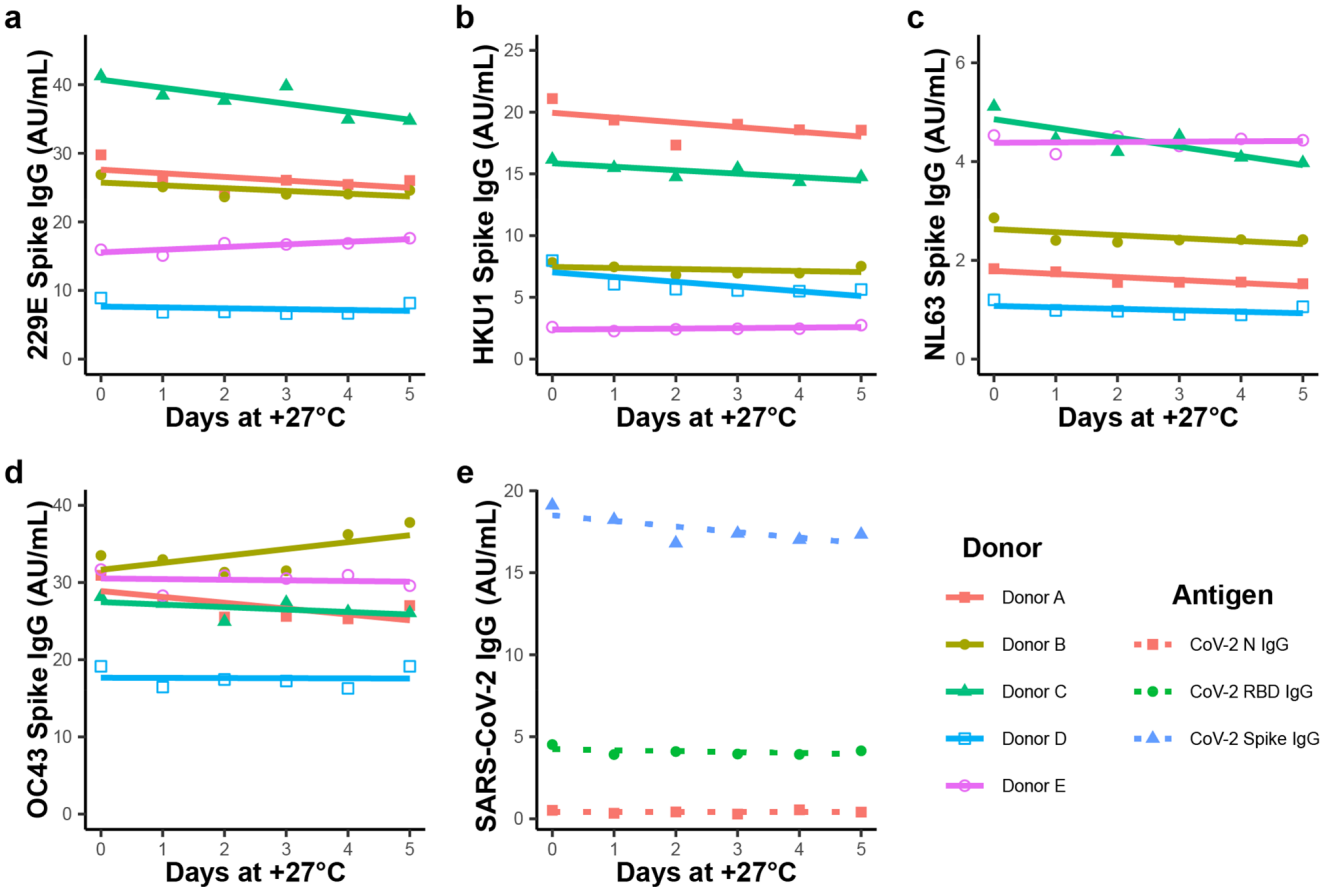
Room temperature stability of saliva. Five donors provided self-collected saliva. For each donor, one aliquot was refrigerated promptly at + 4 °C, and 6 aliquots were incubated at + 27 °C. To replicate delays in transport to cold storage at a laboratory, an aliquot of saliva was transferred from the + 27 °C incubator to the + 4 °C refrigerator daily for 6 days. (**a**–**d**) At the end of 6 days, samples were assayed for levels of IgG antibodies to the spike proteins of the four circulating coronaviruses. (**e**) Only Donor B had detectable IgG antibodies for SARS-CoV-2 N and spike proteins. Overall, prolonged storage at room temperature for 5 days did not significantly alter antibody measurements relative to prompt refrigeration. See supplementary materials for similar graphs showing stability at + 4 °C relative to < − 70 °C.

We measured the stability of antibodies in self-collected liquid saliva samples collected by a passive drool method, and stored without any additional preservatives or stabilizers. Antibody concentrations, as measured by indirect serology using a panel of coronavirus antigens, was followed over 6 days of storage at + 27 °C or + 4 °C, a time scale longer than the expected 1 to 3 business-day delivery time for US Postal Service Priority Mail. The measured concentrations are provided in Fig. 1. As shown in Fig. 1a–d, all five donors showed IgG reactivity to spike proteins of circulating coronavirus (229E, HKU1, NL63, and OC43), as would be expected based on analogous serum data for adult subjects^23^. The figure shows that the levels of antibody against these four antigens were stable to sample storage for at least 5 days at + 27 °C, which is consistent with previous reports^24^. Levels of salivary antibodies for the four circulating coronaviruses declined on average 0.2% to 2% per day over the first 5 days at + 27 °C. One sample (from Donor B) exhibited a drastic decline in concentration at day 6, but all the other samples remained stable at + 27 °C for 6 days. None of the samples showed significant changes in antibody concentrations against the circulating coronavirus antigens when stored at + 4 °C over 6 days relative to storage at − 70 °C (Supplementary Fig. 2).

Of the five samples used in stability studies, only one donor (Donor B) had reactivity to SARS-CoV-2 antigens. Figure 1e shows that this sample had elevated levels of antibodies against SARS-CoV-2 spike and RBD, and also shows that the stability of the antibodies to these antigens was comparable to the stability of the antibodies against the circulating coronavirus antigens (Fig. 1a–d). Notably, for Donor B the measured antibodies against the CoV-2 antigens exhibited the same drop in concentration after day 5 that was observed for the antibodies against the circulating coronavirus antigens. This result suggests that levels of antibodies to the circulating coronaviruses (which should be elevated in most adults) could be used as a sentinel indicator of sample degradation.

We also measured the stability of the same 5 saliva samples to repeated freeze-thaw cycles and found no significant change in the measured concentrations after 3 or 5 cycles (see supplementary materials). Based on the observed stability of salivary antibodies to freeze thaw and extended exposure at + 27 °C, for the remaining experiments we continued to collect saliva using our passive drool method without antimicrobials or protein stabilizers, and to freeze samples upon receipt until batch testing.

### Received specimens and surveys of COVID-19 exposure

In our first model of community-wide serology testing, we received specimens from 132 MSD employees, or their family members, who self-collected saliva and/or finger-stick samples and dropped them off anonymously at room-temperature drop-off boxes placed at MSD facilities, from which samples were collected once a day. Matched saliva and finger-stick blood was provided by 125 of these donors. Six donors only provided saliva samples. The tube of saliva for one donor with a PCR confirmed diagnosis of COVID-19 arrived empty with a partially detached cap, and was not included in the analysis. The absorbent material contained saliva within the biohazard bag as intended. Except for the one empty specimen tube, all returned specimens were of sufficient quantity to allow for analysis. All were packaged according to the provided instructions.

Table 1 summarizes responses to the survey on COVID-19 diagnosis, exposure, and symptoms. The majority of study donors (n = 107; 81%) were classified as “Presumed Naïve” (PN), since they reported no symptoms of COVID-19, household exposure, or confirmed diagnosis. Six donors (4.5%) had been diagnosed with COVID-19 by PCR testing (PCR +). All PCR + donors had received a positive PCR result at least 30 days prior to submission of saliva samples. An additional 14 donors (10.6%) did not report a definitive positive PCR result, but reported other risk factors that suggest a risk of prior infection. Of these possibly non-naïve (PNN) donors, seven (5.3%) reported living with someone who had been diagnosed with COVID-19 via a PCR test more than 30 days earlier, including three who reported having symptoms consistent with COVID-19 (household exposure with symptoms; HEWS) and four without symptoms of COVID-19 (household exposure with no symptoms; HENS). Another seven of the PNN donors (5.3%) did not report a positive COVID-19 test or household exposure, but reported recent flu-like symptoms. Five donors (3.8%) did not return a survey with their specimens (NS = no survey), and were excluded from the analyses.

**Table 1:** Diagnosis, household exposure, and symptoms of SARS-CoV-2 infection among donors of self-collected finger-stick blood and saliva. Based on responses to anonymous surveys, study donors were classified into six groups.

SARS-CoV-2 Exposure Category	Positive PCR Test	Household Exposure	COVID-19 Symptoms	Number of Donors
**Presumed Naïve (PN)**	No	No	No	107
**Positive COVID-19 PCR (PCR +)**	Yes	Yes or No	Yes or No	6
**Possibly Non-Naïve (PNN)**				
Household exposure with symptoms (HEWS)	No	Yes	Yes	3
Household exposure with no symptoms (HENS)	No	Yes	No	4
COVID-19 Symptomatic (CS)	No	No	Yes	7
**No Survey (NS)**	No response	No response	No response	5

### Quality assessment of self-collected specimens

Given that the samples were self-collected without supervision, we sought to verify that specimens were collected and delivered in a usable form without significant deterioration in antibodies, or high levels of food particles or phlegm. Quality of saliva samples was assessed by visual inspection and by measuring salivary antibody content. Saliva samples differed widely in appearance and volume. Volumes ranged from approximately 0.2 mL to 2 mL. Appearances ranged from clear to foamy and in some cases grey. All samples could be pipetted readily, and none were predominantly phlegm.

We verified that samples contained expected levels of immunoglobulins as a basic indicator of sample integrity. Median concentrations of total immunoglobulin in reconstituted finger-stick blood were 150 µg/mL, 30 µg/mL, and 53 µg/mL for IgG, IgM, and IgA, respectively (see supplementary materials for additional summary statistics). Based on the capacity of the swab (10 µL of blood), the volume of extraction buffer (200 µL), an estimated swab extraction efficiency (~ 80%) and a typical hematocrit (~ 50%), multiplying these concentrations by a conversion factor of roughly 50 (200 µL/(10 µL × 50% × 80%)) should provide an estimate of the antibody concentrations in the plasma or serum fraction of the original blood sample. This conversion provides estimated plasma/serum concentrations of 7.5 mg/mL for IgG, 1.5 mg/mL for IgM and 2.7 mg/mL for IgA. These values are comparable to the median measured concentrations of immunoglobulins in serum of 11 mg/mL, 1.3 mg/mL, and 2.4 mg/mL for IgG, IgM, and IgA, respectively, as reported by Gonzalez-Quintela et al*.* using a commercial nephelometry assay on a BN-II device (Dade Behring, Marburg, Germany)^25^.

Median concentrations of total salivary immunoglobulin were 1.5 µg/mL, 2.9 µg/mL, and 83 µg/mL for IgG, IgM, and IgA, respectively. Concentrations of total salivary immunoglobulins were similar to published ranges measured using different assays and collection methods (IgG range = 0.4–93 µg/mL^26^; IgM = 0.5–13.0 µg/mL^27^; IgA = 50.2 ± 19.1 µg/mL^28^). Median concentration of salivary IgG was 100-fold lower than measured in our diluted finger-stick blood samples and 7300-fold lower than reported for undiluted serum. The variation in total immunoglobulin concentrations across donors was higher in saliva than in finger-stick blood. The ratio of the 75th percentile to the 25th percentile for IgG levels was 4.7 for saliva compared to a ratio of 1.6 for finger-stick blood.

As an additional assessment of sample quality, we measured the levels of antibodies to spike proteins for circulating coronaviruses. Prior infection with these endemic viruses is common^29,30^, and we expected that all donors would have high levels of antibodies to at least one of the four circulating coronaviruses on the panel^31^. Consistent with the levels of total immunoglobulin in finger-stick blood and saliva relative to serum discussed above, serum levels of antibodies to circulating coronaviruses were on average 51-fold and 2800-fold higher than in finger-stick blood and saliva, respectively (Supplementary Table 4).

Of the 125 donors providing matched saliva and finger-stick blood samples, two PN donors had normal levels of total immunoglobulin, and antibodies against the circulating coronaviruses in their blood sample, but not in saliva. One of these donors showed strong IgG reactivity to 229E Spike in finger-stick blood (850 AU/mL; above the 75th percentile), but showed background IgG reactivity to 229E Spike in saliva. The other donor showed strong IgG reactivity to OC43 Spike in finger-stick blood (1500 AU/mL; above the 75th percentile), but showed background IgG reactivity to OC43 Spike in saliva. This result indicates a likely issue in the collection and/or handling of these samples, but also suggests that measurements of total immunoglobulin levels, or measurements of antibodies against high prevalence endemic viruses such as the circulating coronaviruses, could be used to identify problematic samples. Saliva from these two donors was excluded from analysis of SARS-CoV-2 antibody responses.

### Establishing the normal ranges for reactivity to CoV-2 antigens among likely non-infected controls

**Figure 2:**
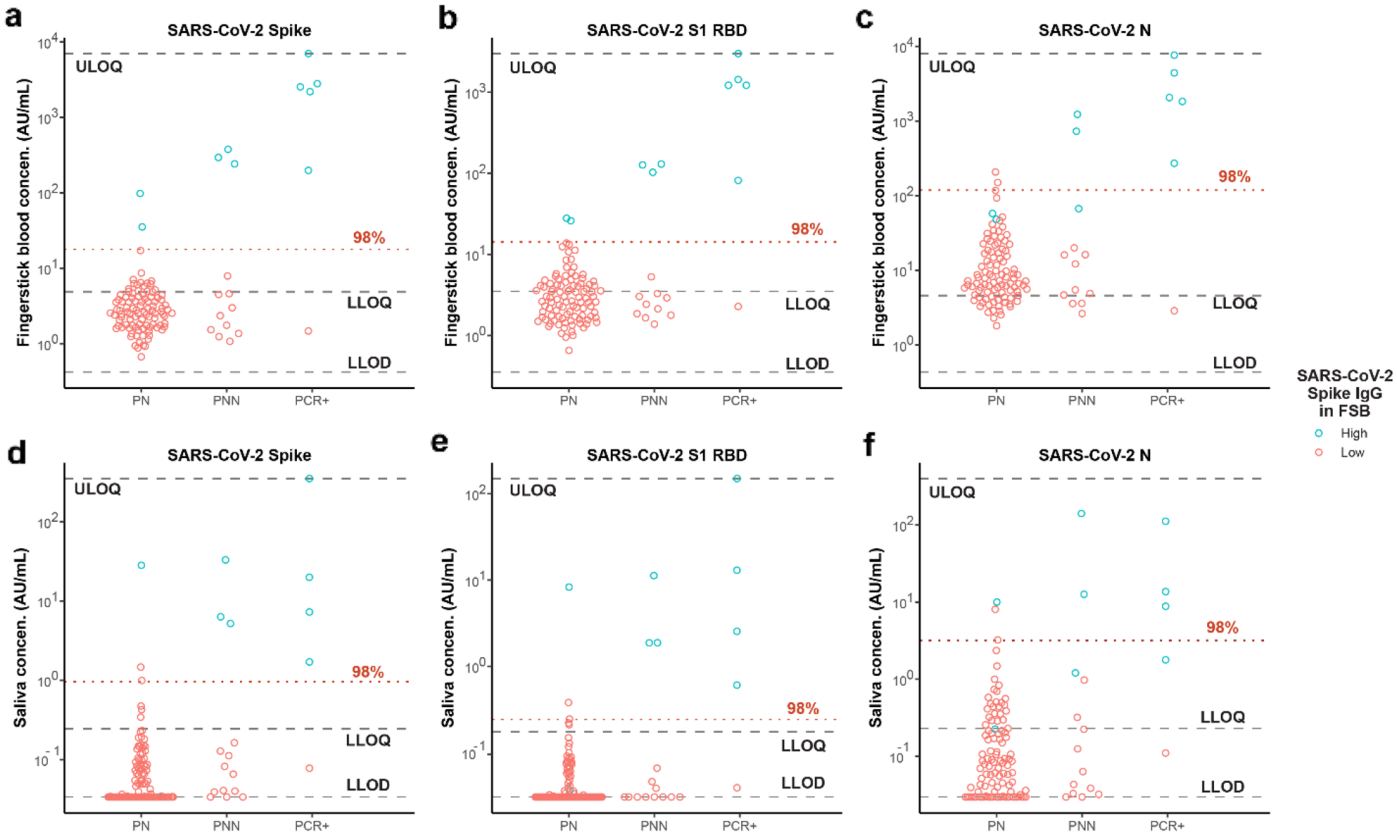
Reactivity to SARS-CoV-2 antigens in self-collected finger-stick blood (FSB) and saliva. Concentrations of anti-SARS-CoV-2 IgG antibodies are plotted for donors who have been grouped based on survey responses about CoV-2 infection, exposure, and symptoms as detailed in Table 1. Dashed lines indicate assay sensitivity and quantitation (LLOD = lower limit of detection; LLOQ = lower limit of quantitation; ULOQ = upper limit of quantitation). The red dotted line labeled “98%” is drawn at the threshold set at the 98th percentile for the presumed naïve (PN) donors. Blue indicates donors whose IgG levels in finger-stick blood exceeded the threshold for SARS-CoV-2 spike.

Figure 2 shows the measured concentrations of antibodies to the SARS-CoV-2 antigens in finger-stick blood and saliva from all donors. We established the normal ranges for the SARS-CoV-2 serology assays using the samples from the 107 study donors who were unlikely to have had prior infection with COVID-19 (the PN group). Preliminary threshold values for classifying individuals with prior SARS-CoV-2 infections were determined based on the 98th percentile for the normal range. This approach provides a tolerance for a 1% to 2% rate of undetected asymptomatic infection in this Presumed Naïve group. Overall seropositivity at the time of this study is estimated at 4.4%, based on a study of health care personnel without patient contact within the same metropolitan area performed at approximately the same time^32^. Since half of SARS-CoV-2 infections are thought to be asymptomatic^33^, the expected prevalence of seropositivity resulting from asymptomatic infection is approximately 2%.

At the selected dilution, most of the saliva samples from the PN group were below the LOD for reactivity to the SARS-CoV-2 spike and RBD antigens. A higher percentage of these saliva samples had detectable reactivity against SARS-CoV-2 N antigen, which may result from the presence of cross-reactive antibodies originally induced by other coronaviruses. The selected classification thresholds for extracted finger-stick blood were 119 AU/mL, 14 AU/mL, and 18 AU/mL for IgG against SARS-CoV-2 N, RBD, and Spike, respectively. The selected classification thresholds for saliva were 3.2 AU/mL, 0.24 AU/mL, and 0.96 AU/mL for IgG against SARS-CoV-2 N, RBD, and spike, respectively.

### Reactivity to CoV-2 antigens in finger-stick blood samples

Figure 2 shows the measured levels of IgG antibodies against the three SARS-CoV-2 antigens (spike, RBD and N) in finger-stick samples, relative to the selected thresholds. By definition, as the thresholds were defined as the 98th percentiles for the PN group, 2% (2 of 107) of the PN samples were classified as positive by each assay. Each of the three assays identified 5 of 6 of the PCR + (confirmed positive) donors. The PCR + donor that was classified as negative reported an asymptomatic COVID-19 diagnosis more than 30 days previously, but had no significant reactivity to any SARS-CoV-2 antigen for any isotype. This individual had total immunoglobulin levels within the normal range as well as normal reactivity to circulating coronaviruses. A humoral response in this individual may have waned or not developed^34^. For the PNN participants that were considered potentially non-naïve to SARS-CoV-2 based on symptoms and/or household exposure (the CS, HEWS and HENS groups), the spike and RBD assays classified 3 of 14 as positive (2 symptomatic and 1 asymptomatic donor with household contacts). The N assay also classified 2 of these 3 as positive, the third falling just under the threshold. Interestingly, none of the donors who reported possible COVID-19 symptoms, but no confirmed diagnosis or household exposure, had elevated antibody levels to SARS-CoV-2 antigens. This suggests that non-specific symptoms may be unreliable indicators of past infection. Alternatively, antibody levels may have waned faster in mild cases for which donors did not seek testing^35,36^.

As a guide for tracking samples across the different assays shown in Fig. 2, blue symbols are used to highlight samples provided by donors whose IgG levels in finger-stick blood exceeded the threshold for spike protein. Among the confirmed or possibly infected individuals (PCR + and PNN groups), the same 8 finger-stick samples showed elevated reactivity to all three SARS-CoV-2 antigens, although one of the samples was just under the threshold for the N assay.

### Reactivity to CoV-2 antigens in saliva

Figure 2 also shows the measured levels of IgG antibodies against the three SARS-CoV-2 antigens (spike, RBD and N) in saliva samples. For samples from donors that were confirmed or possibly infected (PCR + and PNN groups), measurements of IgG against spike and RBD proteins in finger-stick blood and saliva samples provided complete agreement in classification. Measurement of IgG against the N protein performed similarly except for one PCR + donor who obtained a positive result for N in blood but not saliva. The two PN samples that were classified as positive varied for the different assays and sample types, although there was one PN donor that was classified as positive based on IgG against spike and RBD in blood, and spike, RBD and N in saliva, suggesting this individual may have had an asymptomatic infection.

**Figure 3:**
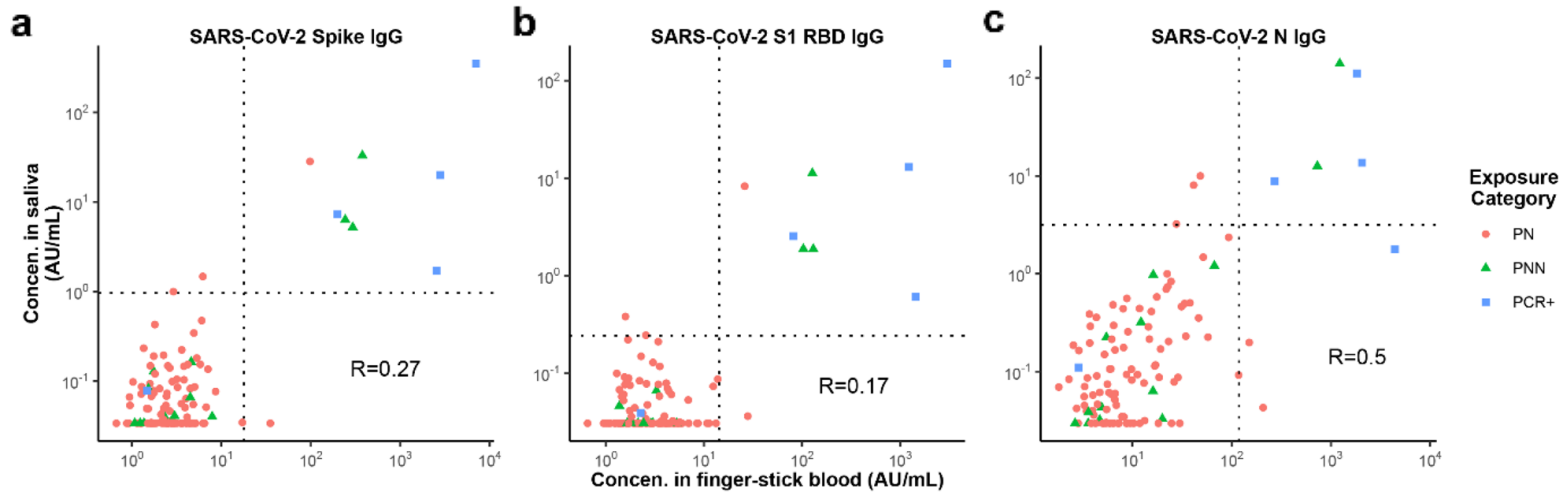
Correlation in reactivity to CoV-2 antigens measured in self-collected saliva versus finger-stick blood. Levels of IgG antibodies were measured in matched saliva and finger-stick blood provided by the same donors. Dotted lines indicate the selected classification thresholds. For each figure, the lower left quadrant contains samples that are within the range of non-specific reactivity for both saliva and finger-stick blood. The upper right quadrant are samples with high reactivity for both saliva and finger-stick blood.

Correlations between the levels of antigen-specific IgGs in saliva and blood samples are shown in Fig. 3. While the agreement between the two matrices for classification was strong, their correlation in levels was only moderate (R = 0.25; *p* = 0.005), which is consistent with another study^17^. In 97.5% of donors with matched saliva and finger-stick blood, saliva and finger-stick blood measurements were concordant for classification of SARS-CoV-2 spike IgG and SARS-CoV-2 S1 RBD IgG levels as high or low relative to their matrix-specific thresholds (Cohen’s κ = 0.83; *p* = 8.4e-18). 112 donors had low levels of SARS-CoV-2 Spike IgG for both saliva and finger-stick blood, and 8 donors had high levels of SARS-CoV-2 Spike IgG in both saliva and finger-stick blood. For the three discordant cases, two donors were slightly above the saliva threshold, and one donor was slightly above the finger-stick blood threshold. The concordance for the SARS-CoV-2 N IgG assays was 95.1% (Cohen’s κ = 0.64; *p* = 2.5e-6).

**Figure 4:**
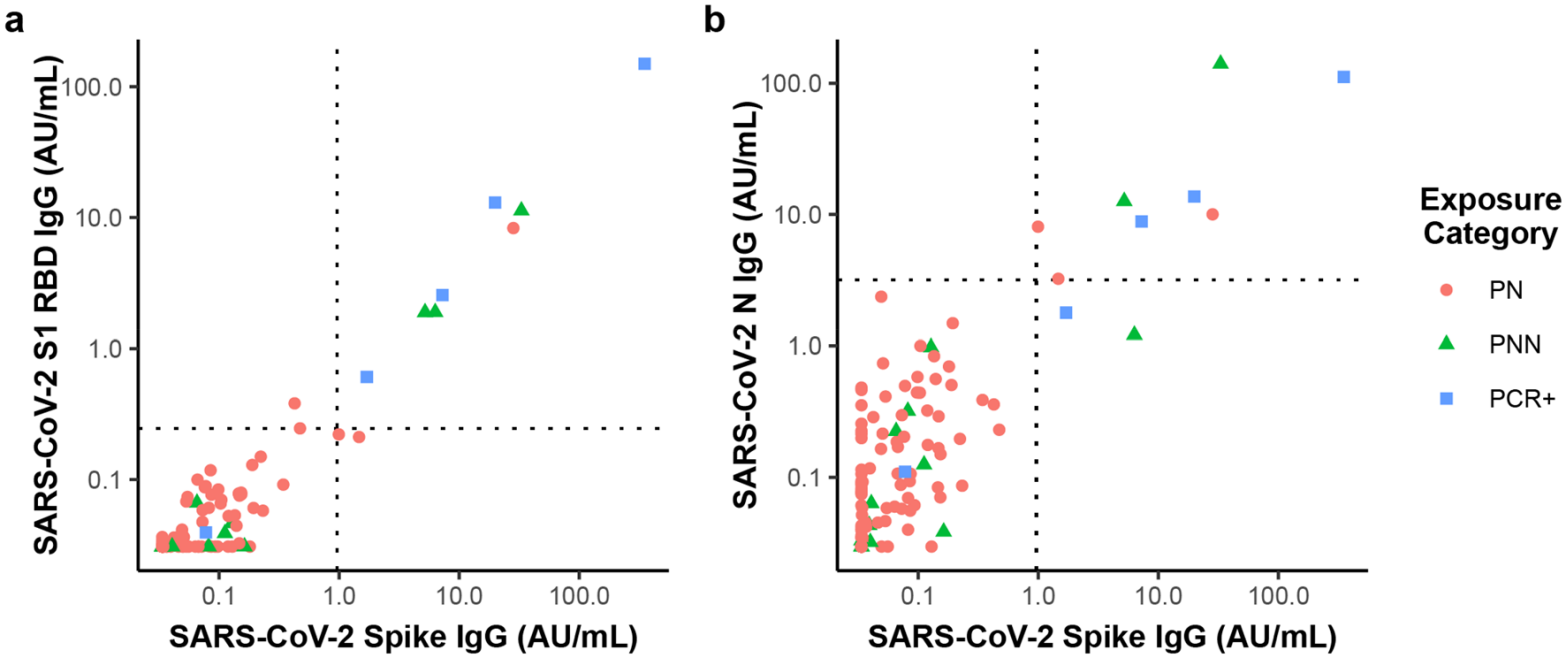
Correlation in salivary IgG levels for CoV-2 spike, RBD, and N antigens. Levels of salivary IgG antibodies to the full length spike protein are highly correlated with IgG antibody levels for (**a**) RBD of the spike protein and (**b**) N antigen. Dashed lines indicate the selected classification thresholds set at the 98th percentile of saliva from PN donors who report no COVID-19 diagnosis, recent symptoms, or household exposure to COVID-19.

The salivary levels of antibodies to the full-length spike and RBD antigens were highly correlated (Fig. 4). Absolute signals for the full-length spike were higher than for the RBD antigen, which is expected since antibodies to the RBD are a subset of those binding to the full-length spike. The salivary levels of antibodies reactive with the N antigen were moderately correlated with antibodies for spike and RBD.

**Figure 5:**
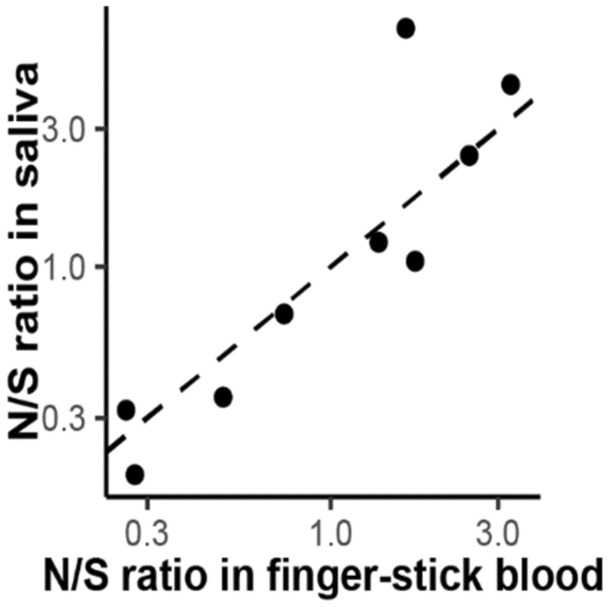
Relative reactivity to the CoV-2 nucleocapsid (N) and spike (S) antigens measured in finger-stick blood and saliva from subjects whose levels of anti-spike IgG exceeded the threshold. The ratio of IgG levels to the N and S antigens (N/S ratio) ranged from 0.12 to 4.3 , and was highly correlated in matched finger-stick blood and saliva provided by the same donors (Spearman coefficient = 0.95, *p* = 0.001). The dashed line has slope of 1 and represents the expected correlation.

Because the relative immune responses to N versus S may be a clinically significant indicator of immune response^37^, we correlated the ratio of anti-N to anti-S levels measured in finger-stick blood versus saliva (Fig. 5). We found a strong correlation in the N to S ratio (R = 0.95; *p* = 0.001), which indicates that quantitative salivary measurements can be used to compute this ratio equivalently to finger-stick measurements.

### Transport of saliva through the mail

**Figure 6:**
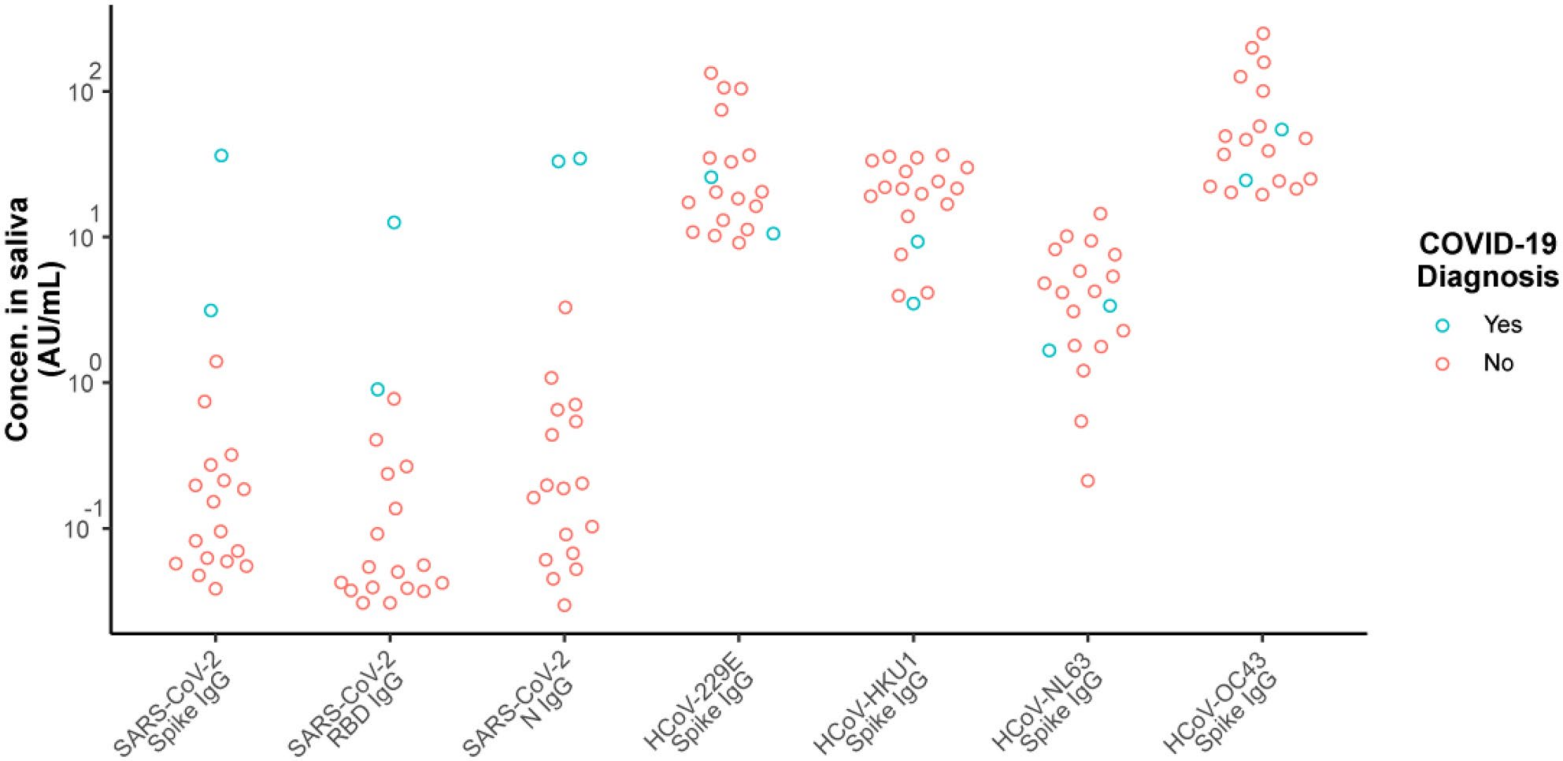
Salivary IgG antibodies measured in self-collected saliva returned via mail. Saliva collection kits were sent via the mail to individuals who requested a kit in response to an online recruitment posting. Donors provided self-collected saliva according to written instructions and completed a survey about COVID-19 testing, which were returned to MSD via a prepaid mailer. Nineteen samples were received, and tested for IgG antibodies to SARS-CoV-2 and circulating coronaviruses. Two donors (shown in blue), who reported a positive COVID-19 PCR test at least 30 days prior to providing a sample, had IgG anti-SARS-CoV-2 antibodies. All donors had levels of antibodies to circulating coronaviruses within the range of levels measured in samples within the local cohort.

A primary purpose of our study was to assess the utility of self-collected samples for large scale serology testing. Due to the excellent stability of salivary antibodies at room temperature and without preservatives, we wondered whether saliva could be transported through the mail. We performed a pilot study where donors mailed saliva specimens to Gaithersburg, Maryland from Oklahoma. We identified no significant logistical challenges in our pilot study. Using only written instructions provided in the kits, donors were able to collect samples and return them according to UN3373 regulations. Specimens (n = 19) showed expected levels of antibodies to circulating coronaviruses as shown in Fig. 6. Although mailed-in samples spent up to two weeks in transit, the antibody concentrations for the circulating coronaviruses in saliva sent through the mail were not lower than for locally collected samples (one-sided Mann–Whitney *p* ≥ 0.99). Anti-SARS-CoV-2 antibodies were detected only in the two samples from individuals who responded that they had previously been infected by SARS-CoV-2. Overall, this pilot study demonstrates the feasibility of a “spit and mail” test for salivary antibodies.

## Discussion

We measured anti-CoV-2 antibodies in finger-stick blood and saliva that anonymous donors self-collected in two models of community-wide serology testing. In the first, collection kits were locally picked up and dropped off at a return box. In the second, kits were distributed and returned entirely through the mail. This study advances methods for serosurvillance by directly comparing self-collected finger-stick blood against saliva self-collected with a simple kit designed to maximize scalability and minimize downstream sample processing and handling. In prior studies^17,38^, saliva was collected under supervision in research settings using devices that are not FDA approved and/or require secondary containers to be compliant with UN3373 category B shipping requirements. This study collected samples using FDA registered devices that can be sent through the mail. Moreover, this study employs the use of quantitative, multiplexed immunoassays that can be automated to achieve the high-throughput required for population-level testing.

Antibody levels measured in finger-stick blood have previously been shown to correlate with serum measurements^5–8^. Our most significant finding is that self-collected saliva provides information similar to finger-stick blood even when saliva is collected without supervision or in-person training and even after being at ambient temperature for hours to days without preservatives. Consistent with prior studies that report a correlation between antibody levels in serum and saliva^13,14^, we observed very high concordance between self-collected saliva and finger-stick blood in the identification of individuals with IgG reactivity to the RBD and full-length forms of CoV-2 spike protein.

Studies on the origin of IgG in saliva have led to models in which the bulk of salivary IgG originates from the blood stream due to transudation or bleeding from the gingival tissue^39–41^. Although salivary IgG levels reflect serum IgG levels, salivary IgG levels appear to be affected by other factors that make the range of IgG levels measured in saliva broader than the range for serum. The degree of correlation between finger-stick blood and saliva measurements (Fig. 3) depends on variations among individuals in the rate of antibody transit into the mouth, the rate of saliva flow diluting the antibody, and possibly other factors associated with the degree of compliance with instructions for sample collection such as delaying collection after eating or drinking^26^. The almost perfect agreement we observed in the classification of serostatus using saliva and finger-stick blood suggests that the difference in the observed antibody activity in positive subjects vs. negative controls is large enough to compensate for the increased variability in saliva samples. We found that the ratio of anti-N antibodies to anti-S antibodies in saliva and finger-stick blood (Fig. 5) correlates more strongly than the ratio of the absolute concentrations (Fig. 3), showing that the effect of variations among donors in salivary flow rates and rates of antibody transit can be reduced through normalization approaches.

Based on results of stability testing and testing specimens collected locally, we explored a more versatile strategy to distribute kits and return specimens through the mail. Our pilot study demonstrated that saliva can be self-collected, packaged, and mailed in accordance with UN3373 and CDC guidelines using only written instructions. The success of the pilot study highlighted the importance of logistical insights such as using the most compact packaging that satisfies regulatory requirements. The compact dimensions allowed for easy return of packages from diverse locations where return of large packages is not convenient. One lesson learned in the development of our approach was that postal workers did not always pick-up packages left in mailboxes, especially in locations with multiple boxes, and no specially designated outgoing mail slot. We found that this problem could be effectively addressed by attaching a removable label indicating the package as outgoing.

Despite limitations inherent in anonymous sample collection that preclude longitudinal sampling and confirmation of COVID-19 testing with medical documentation, we showed that MSD’s multiplexed immunoassays have essential features for population-level testing. Sample collection can be done without in-person training or supervision. Sample transport requires no refrigeration. Processing is minimal and requires no specialized equipment. Measurements of salivary antibodies were performed on an automated robotic system capable of supporting large-scale testing. Stability and ease of use appear sufficient for a “spit and mail” test. Follow-up testing with well-annotated specimens from a large number of COVID-positive donors is needed to determine the clinical sensitivity and specificity of the assay.

A convenient, non-invasive serology test may be generally useful for epidemiologic studies^42^. The combination of asymptomatic infection and shortages of diagnostic testing complicates accurate retrospective determination of infections when studying viral transmission within households and other communities. Thus far, serosurveillance studies have relied on testing of blood at dialysis or blood donation centers^43–47^. A “spit and mail” test may reach a representative cross-section of the population, which is a limitation of serosurveillance conducted using blood samples collected from a non-random subset of the population. If antibody levels are shown to be correlates of immunity, further studies are warranted to assess the practicality of serologic testing of self-collected saliva for screening individuals who may benefit from a booster.

## Methods

### IRB approval of anonymous sample and data collection

IRB approval was obtained for two protocols to collect finger-stick blood and saliva anonymously, as well as to survey SARS-CoV-2 exposure, COVID-19 risk factors, and demographics. Due to the low risk and anonymity of the study, written documentation of informed consent was waived. In the first protocol (Advarra Pro00043585), volunteers among MSD employees and their family members provided matched finger-stick blood and saliva via self-collection kits. The second protocol (Advarra Pro00045143) expanded saliva collection to adults within the United States, who were recruited via online ads and sent a saliva-collection kit and survey through the mail. Samples were collected from July to October, 2020.

### Self-collection of finger-stick blood

Finger-stick blood was collected using the Mitra collection kit (Neoteryx 100504-A) according to the manufacturer’s instructions. Briefly, a spring-loaded lancet provided with the kit was used to draw blood from a fingertip. Blood was collected onto two swabs, each capable of absorbing 10 µL of blood. The blood dried rapidly on the swabs, which were enclosed in a cartridge and sealed in a bag containing a desiccant. Donors submitted the samples to MSD via drop box.

### Self-collection of saliva

Saliva was collected into a 2 mL screw-cap centrifuge tube (Sarstedt #72.609 with screw cap 65.716.xxx) using the Saliva Collection Aid (SCA; Salvimetrics 5016.02), which is a straw-like device cleared by the FDA for collection of samples from adults and children. The screw cap contained an O-ring in order to be compliant with shipping requirements for Category B Biological Substances (UN3373). Donors were instructed to wait 30 min after eating, drinking, or smoking before drooling into the tube according to the manufacturer’s directions. Donors were instructed to provide saliva without assistance from others. The donors capped their tube, and placed it inside a biohazard bag (VWR 11215-684) containing an absorbent pad (ThermoSafe ZORB66).

Donors enrolled from MSD employees and family members submitted their saliva and finger-stick blood samples together via drop box. Donors recruited by online ads were sent saliva collection kits through the mail. The kits included the collection aid and tube, instructions, and the packing materials needed to mail samples back to MSD. To submit their samples, these donors were instructed to place the biohazard bag containing the tube of saliva in a peel-and-seal cardboard mailer (Stephen Gould; MSD-CFM-SM) bearing a UN3373 label (LabelMaster L380B) and pre-paid shipping label. The COVID-19 taskforce of the USPS reviewed the shipping materials and instructions for packing and mailing the specimens in order to ensure compliance with CDC guidelines and USPS regulations for shipping clinical diagnostic specimens falling under the classification of Category B Biological Substances (UN3373). To support pick-up from home or apartment mailboxes, a removable label was attached to each mailer alerting mail carriers to collect the package.

### Sample receipt and storage

Upon delivery to MSD via a drop box or via USPS Priority Mail, saliva was frozen at ≤ − 70 °C without additional processing. Mitra cartridges were disassembled to remove the swabs. For reconstitution of the dried blood, swabs were placed into 2 mL microcentrifuge tubes containing 200 µL of MSD® Diluent 100 (MSD R50AA) and extracted for 1 h at room temperature with gentle shaking at 700 RPM. After 1 h, the swab was removed and discarded. The microcentrifuge tube containing extracted whole blood was capped and frozen at ≤ − 70 °C.

### Indirect serology

Indirect serology measurements employing electrochemiluminescence (ECL) detection were conducted using kits, reagents and instruments that are commercially available from MSD. On the day of sample testing, saliva and extracted finger-stick blood were thawed at room temperature. Saliva was centrifuged briefly to pull down any food particles or mucus. Prior to analysis, saliva samples were diluted five-fold by combining 20 µL of sample with 80 µL of a sample diluent (MSD Diluent 2). Extracted finger-stick blood was diluted 100-fold by combining 10 µL of sample with 990 µL of a different diluent (MSD Diluent 100).

Diluted samples were assayed in a 96-well plate format using MSD® V-PLEX COVID-19 Coronavirus Panel 2 kits for measuring IgG (K15369U), IgM (K15370U), and IgA (K15371U) antibody responses. Each well of the plates included an antigen array that enabled the multiplexed measurement of antibody responses against nine different coronavirus antigens: four SARS-CoV-2 antigens (the nucleocapsid protein, the spike protein, the spike receptor binding domain (RBD) and the spike N-terminal domain (NTD)) and spike proteins from five other coronaviruses (SARS-CoV-1 and four circulating coronaviruses 229E, HKU1, NL63, and OC43).

The assays were run according to the protocols provided in the assay kit package inserts for serum samples, except for the use of sample diluents and dilution factors (as described above) that were optimized for saliva and finger-stick blood samples. Briefly, the indirect serology protocol involved (i) blocking the 96-well assay plate with a blocking solution, (ii) washing the plate then incubating the diluted samples in the wells of the assay plate to allow antibodies in the samples to bind to the array of immobilized antigens, (iii) washing the plate then incubating the wells with a labeled (MSD SULFO-TAG™ ECL label) anti-human immunoglobulin secondary antibody (anti-human IgG, IgM or IgA) to detect bound antibodies of the selected immunoglobulin type; (iv) washing the plate and adding an ECL read buffer (MSD GOLD™ Read Buffer B); and (v) analyzing the plate on an ECL plate reader (MESO® SECTOR S 600 or MESO QuickPlex® SQ 120). Testing of saliva samples was carried out in an automated fashion using high-throughput automation developed at MSD. Time-to-result was approximately four hours.

For quantitation of antibody responses, an eight-point calibration curve was run in duplicate on all plates and the signals for each antigen were fit to a 1/Y^2^-weighted four parameter logistic (4PL) fit. Samples were run in duplicate and the antibody concentration against each antigen was calculated by back-fitting to the appropriate 4PL fit and correcting for dilution. The concentrations were presented in arbitrary units per mL (AU/mL) that were defined relative to the assigned values of the reference standard. Controls were also run in duplicate on each plate including three serum-based controls (provided with the kit) and two saliva-based controls (pooled normal saliva sourced from Lee Biosolutions spiked with serum from COVID-19 patients).

### Measurement of total antibody levels

Total levels of IgG, IgM, and IgA immunoglobulin were measured using MSD’s Isotyping Panel 1 Human/NHP Kit (K15203D) according to the manufacturer’s directions. Extracted finger-stick blood was run at a dilution of 5000-fold. Saliva was run at a dilution of 1000-fold. Calibration and quantitation were carried out as described above for the indirect serology measurements.

### Saliva stability studies

Stability of salivary antibodies was assessed by comparing antibody levels in saliva stored at different temperatures. Fresh saliva from five donors was aliquoted to create a set of 15 aliquots per donor. Six aliquots were stored at + 27 °C and + 4 °C each, and three aliquots were promptly frozen at ≤ − 70 °C. Over the course of 6 days, one aliquot was transferred daily from + 27 to + 4 °C, where it remained until day 6. Similarly, each day, one aliquot stored originally at + 4 °C was frozen at ≤ − 70 °C through day 6. This generated a set of samples that experienced 1 to 6 days at a higher temperature before being stored at a colder temperature in order to replicate the effect of delayed cold storage. In addition to the timed stability study, two aliquots of the frozen saliva were passed through 3 or 5 freeze–thaw cycles to evaluate the effect of repeated freezing and thawing on measured antibody levels. All stability samples were tested by the indirect serology assay.

### Statistical analysis

Data were processed in Excel. Statistical analysis and graphing were performed in R. Concentrations below the limit of detection (LOD) for an assay were set to the LOD, which was defined as the concentration that generates an assay signal 2.5 standard deviations above the assay background. Concentrations above the top calibrator were set to the top calibrator concentration. Correlations were assessed using the non-parametric Spearman correlation (cor.test function of R stats package) unless otherwise noted. Concordance between measurements in saliva and finger-stick blood was assessed using Cohen’s kappa^48^ (Kappa function of R vcd package). Differences between groups were assessed with the Mann–Whitney test (wilcox.test of R stats package).

## Supplementary Information

Supplementary Information.

## Data Availability

The datasets generated and/or analysed during the current study are available from the corresponding author upon reasonable request.
