# Supplementary material for: Quantitative serology for SARS-CoV-2 using self-collected saliva and finger-stick blood
Source: Sci Rep. 2022 Apr 21;12:6560. doi: 10.1038/s41598-022-10484-6 (PMC9021827; doi:10.1038/s41598-022-10484-6)
Supplement: Supplementary file 1 — Supplementary Information. [file 41598_2022_10484_MOESM1_ESM.docx]

Supplementary Materials for:

**Quantitative serology for SARS-CoV-2 using self-collected saliva and finger-stick blood**

Contents

[Supplementary Figure 1. Mailable kit for self-collection of saliva. 2](#_Toc72940010)

[Supplementary Table 1. Average daily percent declines in salivary IgG antibodies stored at +27° C. 3](#_Toc72940011)

[Supplementary Figure 2. Stability of refrigerated saliva. 4](#_Toc72940012)

[Supplement Figure 3. Robustness to repeated freeze thaws. 5](#_Toc72940013)

[Supplementary Figure 4. Total immunoglobulin levels measured in finger-stick blood and saliva. 6](#_Toc72940014)

[Supplementary Figure 5. IgG antibodies to endemic coronaviruses in self-collected saliva. 7](#_Toc72940015)

[Supplementary Figure 6. Reactivity to endemic coronavirus spike proteins in self-collected finger-stick blood (FSB) and saliva. 8](#_Toc72940016)

[Supplementary Table 2. Immunoglobulin concentrations in finger-stick blood self-collected by donors without confirmed COVID-19 diagnosis, household exposure, or recent symptoms. 9](#_Toc72940017)

[Supplementary Table 3. Immunoglobulin concentrations in saliva self-collected by donors without confirmed COVID-19 diagnosis, household exposure, or recent symptoms. 10](#_Toc72940018)

[Supplementary Table 4. Antibody concentrations for endemic coronaviruses in finger-stick blood, saliva, and serum. 11](#_Toc72940019)

[Supplementary Figure 7. Correlation in reactivity to CoV-2 antigens measured in self-collected saliva versus finger-stick blood. 12](#_Toc72940020)


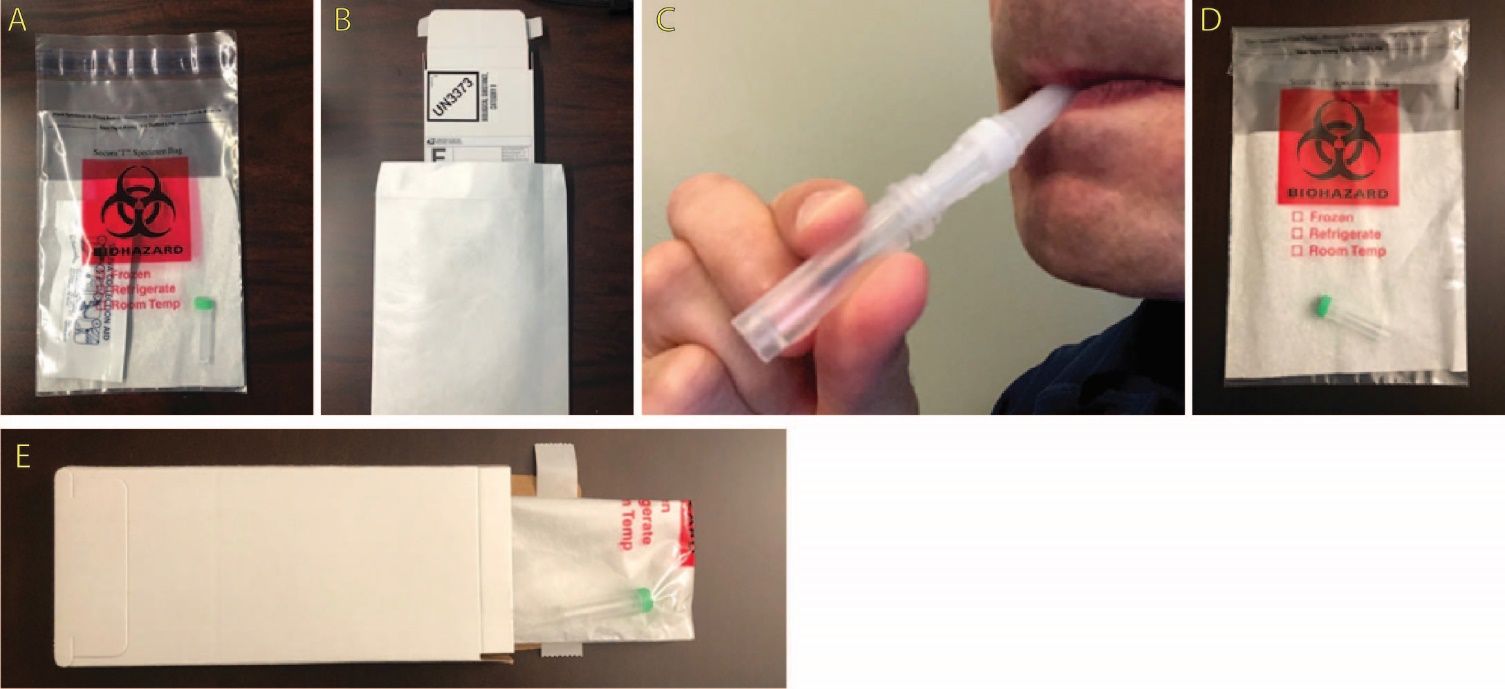


Supplementary Figure 1. Mailable kit for self-collection of saliva. (A) The saliva collection kit consisted of a microcentrifuge tube, O-ring screwtop cap, and saliva collection aid (SCA) placed inside a sealable biohazard bag containing an absorbent material. The kit components were packaged in the same way as they were to be returned into the bag by the study participant. (B) The kit was placed inside of a peel-and-seal cardboard mailer displaying a UN3373 category B label. For mailing packages to study participants, the kit was packed in a Tyvek envelope that accommodated printed instructions, survey, and consent form. Saliva is collected into a microcentrifuge tube (C), which the participants enclose in a biohazard bag (D) and cardboard box (E) prior to mailing back to the laboratory.


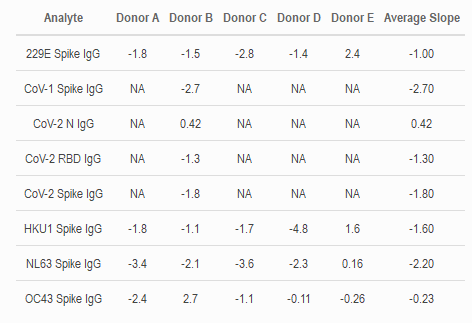


Supplementary Table 1. Average daily percent declines in salivary IgG antibodies stored at +27° C. Fresh saliva from five donors was aliquoted and incubated at +27° C for 0-6 days. Aliquots were transferred daily to a refrigerator and stored at +4° C until testing on day 6. The average daily percent decline over the first 5 days was computed based on the slope of a linear regression line passed through the data points shown in Figure 1 of the main text. The column on the right is the average percent decline for the five donors. Overall, salivary IgG concentrations declined between 1%-2% daily over the first five days post-aliquoting. For one donor, the aliquot stored for 6 days at +27° C had lower levels of antibodies than in aliquots stored +27° C for 5 or fewer days.


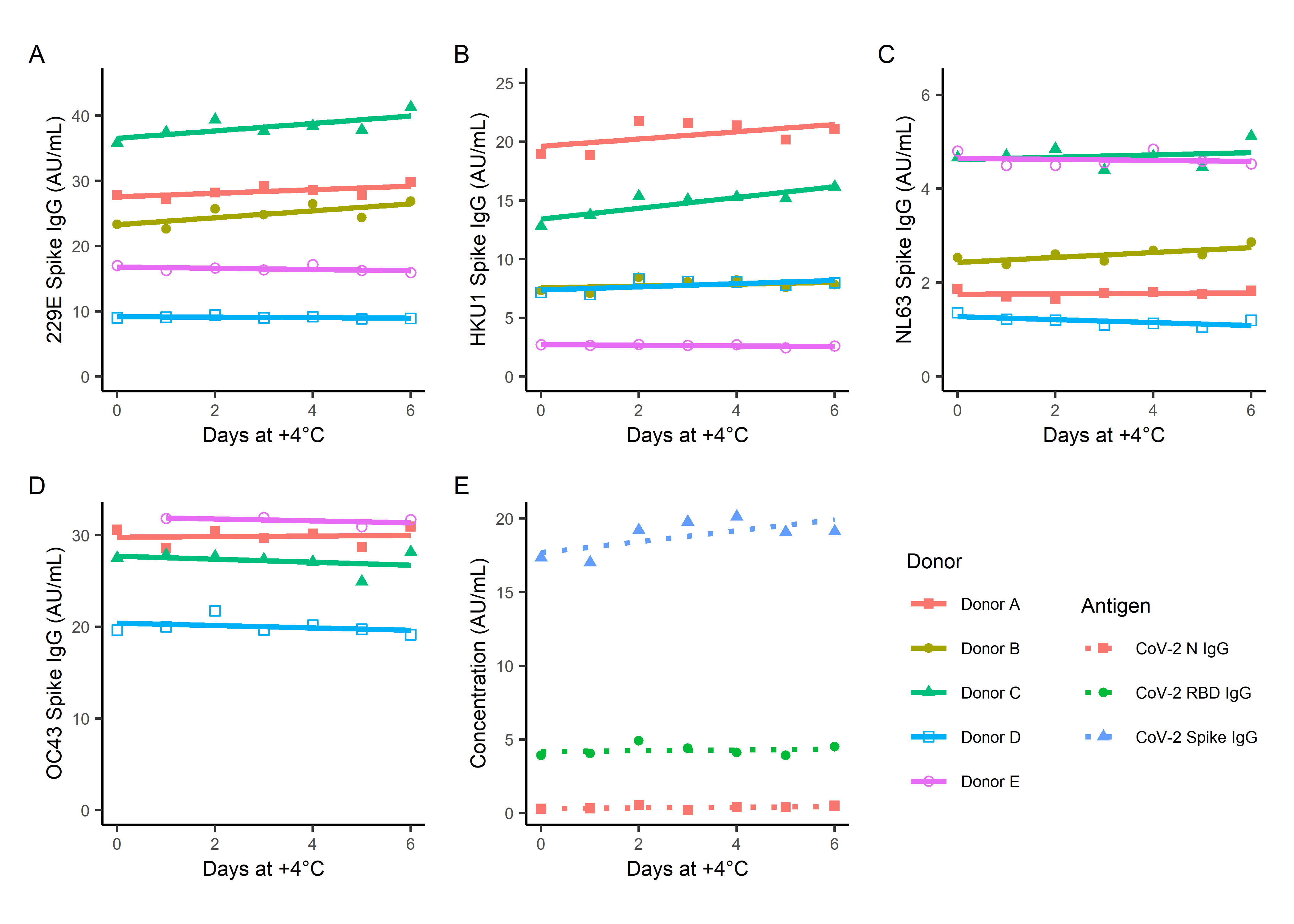


Supplementary Figure 2. Stability of refrigerated saliva. Five donors provided self-collected saliva. For each donor, one aliquot was frozen promptly at <-70° C, and 6 aliquots were refrigerated at +4° C. An aliquot of saliva was transferred from the +4° C refrigerator to the <-70° C freezer daily for 6 days. (A-D) At the end of 6 days, samples were assayed for levels of IgG antibodies to the spike proteins of the four circulating coronaviruses. (E) Only Donor B had detectable IgG antibodies for CoV-2 N and Spike proteins. Overall, prolonged refrigeration did not significantly alter antibody measurements compared to freezing.


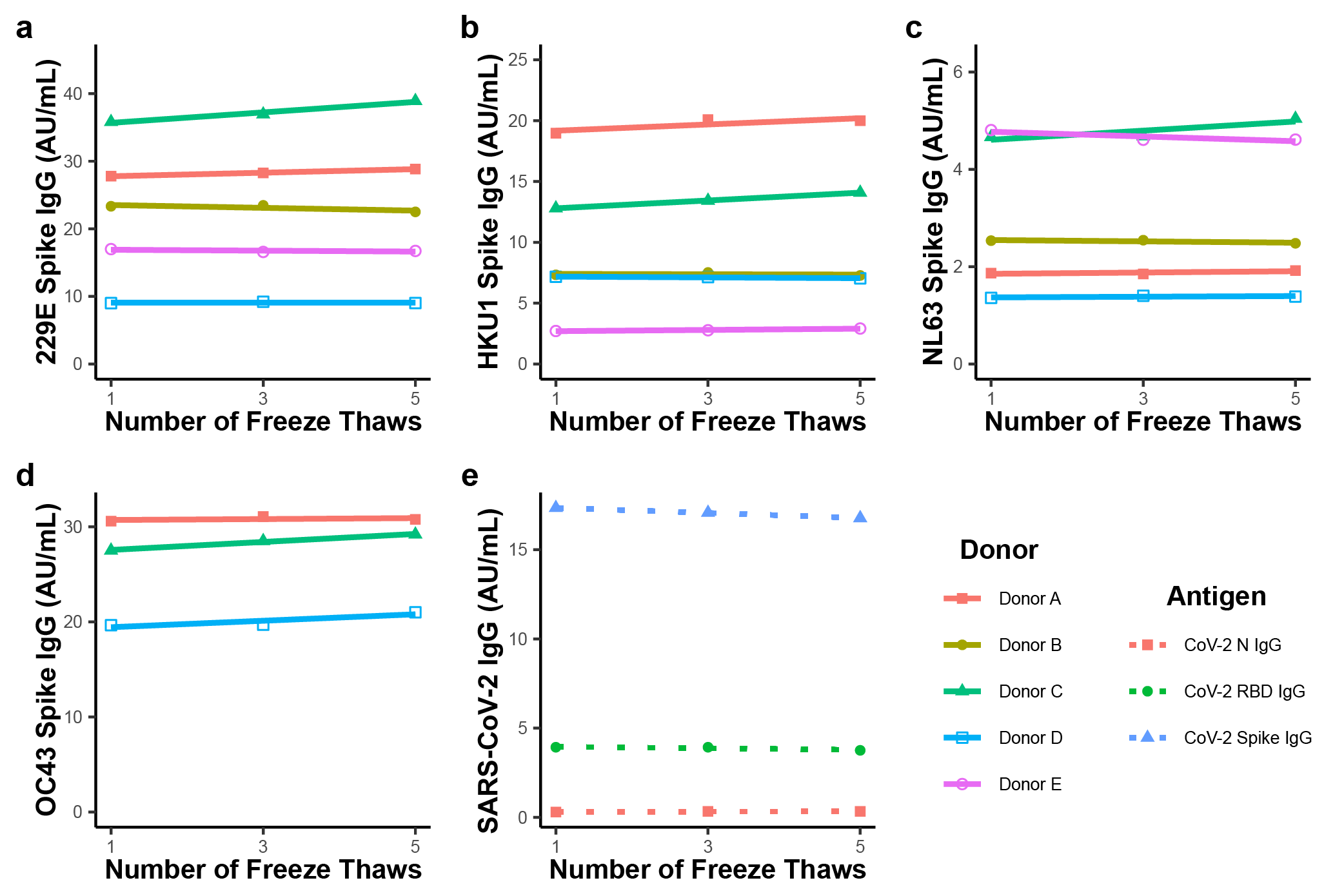


Supplement Figure 3. Robustness to repeated freeze thaws. Aliquots of self-collected saliva from 5 donors were subjected to 1, 3, or 5 freeze-thaw cycles. (a-d) Samples were assayed for levels of IgG antibodies to the spike proteins of the four circulating coronaviruses. (e) Only Donor B had detectable IgG antibodies for CoV-2 N and Spike proteins. Overall, repeated freeze thaws did not significantly alter antibody measurements.


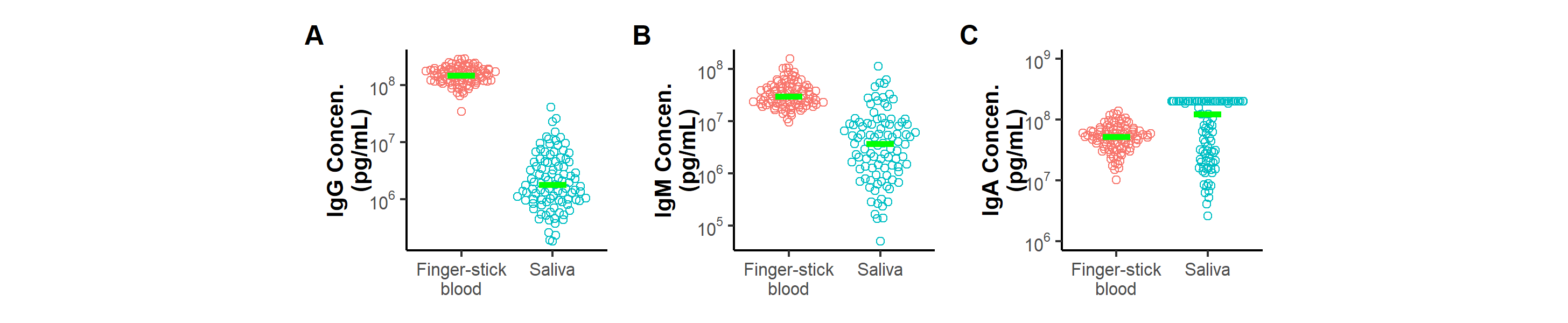


Supplementary Figure 4. Total immunoglobulin levels measured in finger-stick blood and saliva. Finger-stick blood was self-collected via the Mitra device and then reconstituted into diluent. Saliva was self-collected into 2 mL centrifuge tubes using the Saliva Collection Aid (SCA). Plots show the distribution of total immunoglobulin levels measured using Isotyping Panel 1 Human/NHP Kit. Bars indicate median concentrations.


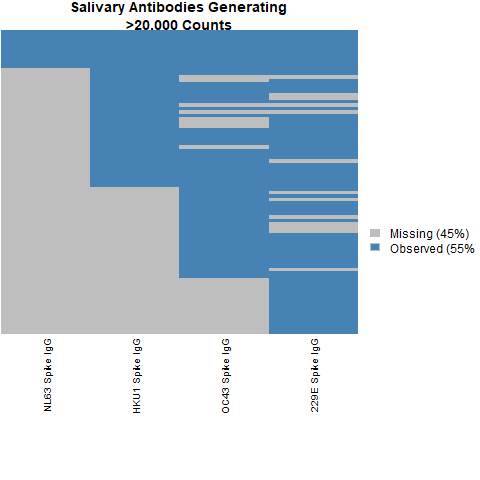


Supplementary Figure 5. IgG antibodies to endemic coronaviruses in self-collected saliva. IgG antibodies to four circulating coronaviruses were readily detected in self-collected saliva. Except for two excluded samples that did not have detectable IgG, IgA, or IgM, all samples contained readily detectable antibodies for at least one circulating coronavirus. Rows correspond to individual saliva samples that were tested for IgG antibodies to the four circulating coronaviruses indicated by the columns. Samples with readily detectable antibodies generating >20,000 counts are colored blue. Samples generating lower signals are colored grey.


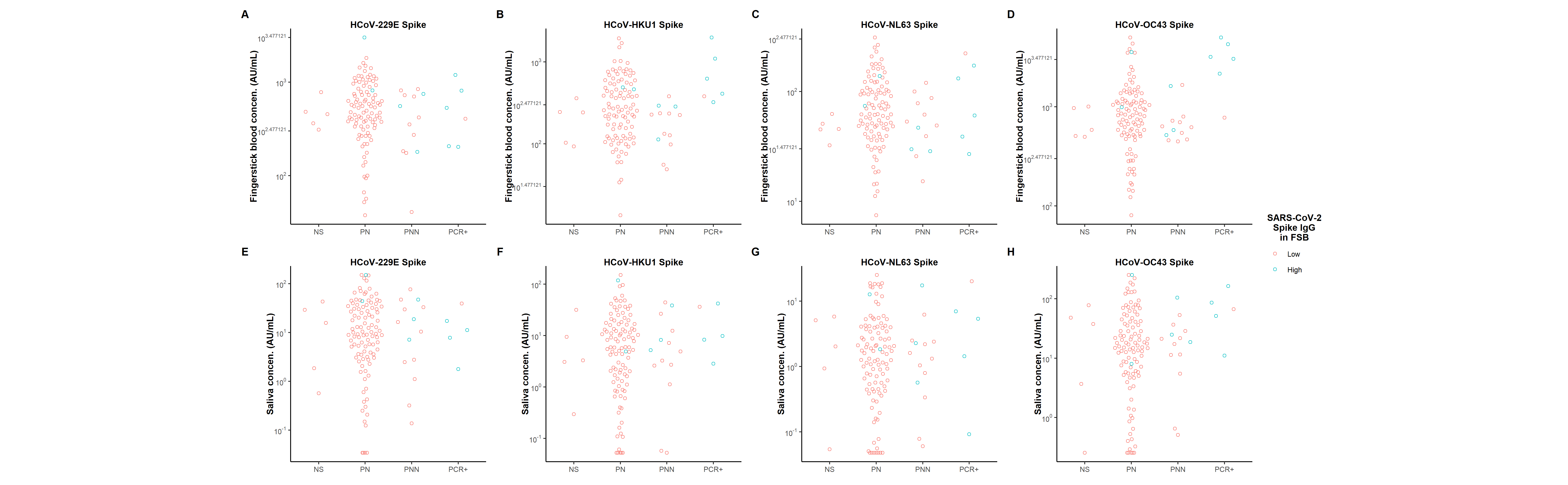


Supplementary Figure 6. Reactivity to endemic coronavirus spike proteins in self-collected finger-stick blood (FSB) and saliva. Concentrations of anti-spike IgG antibodies are plotted for donors who have been grouped based on survey responses about SARS-CoV-2 infection, exposure, and symptoms as detailed in Table 1. Blue indicates donors whose IgG levels in finger-stick blood exceeded the threshold for anti-SARS-CoV-2 spike IgG.


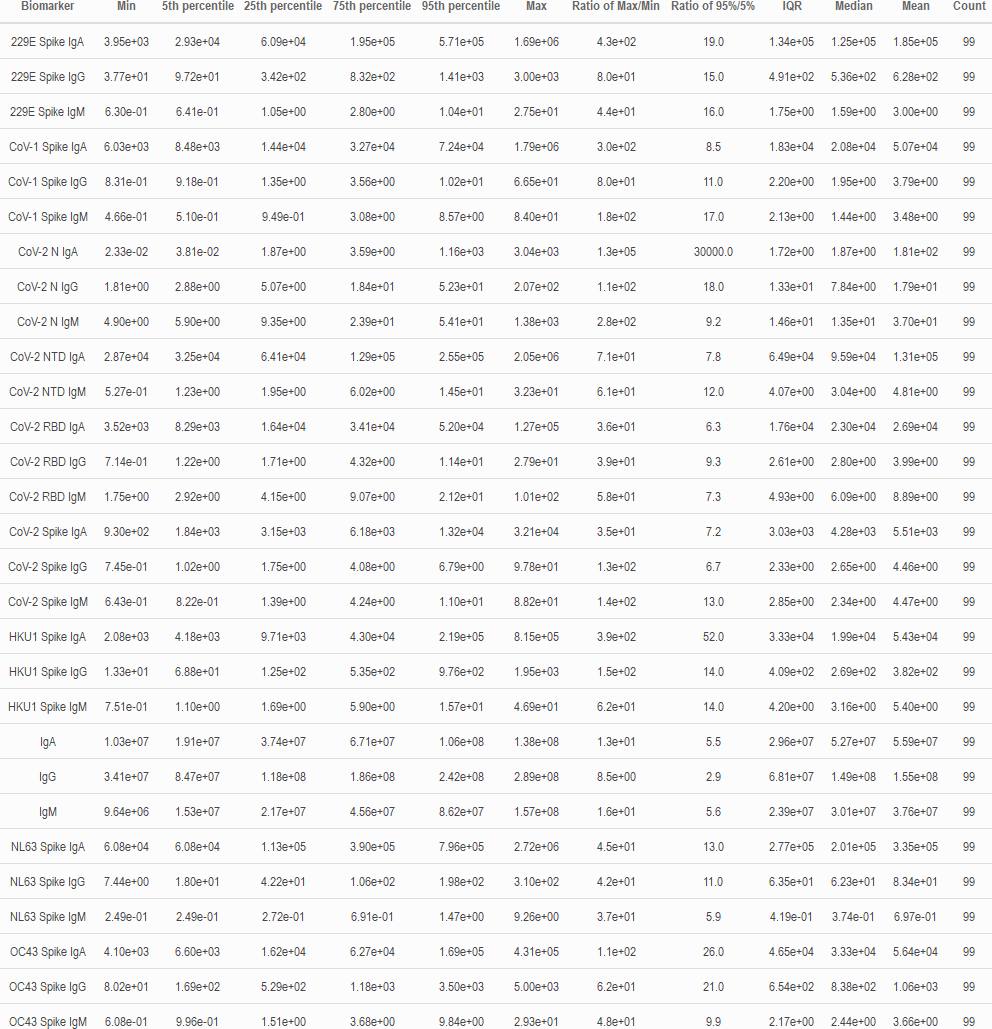


Supplementary Table 2. Immunoglobulin concentrations in finger-stick blood self-collected by donors without confirmed COVID-19 diagnosis, household exposure, or recent symptoms. These measurements were used to establish the upper-limit of non-specific reactivity. Concentrations for total immunoglobulins (IgA, IgG, and IgM) are reported in pg/mL. All other concentrations are AU/mL.


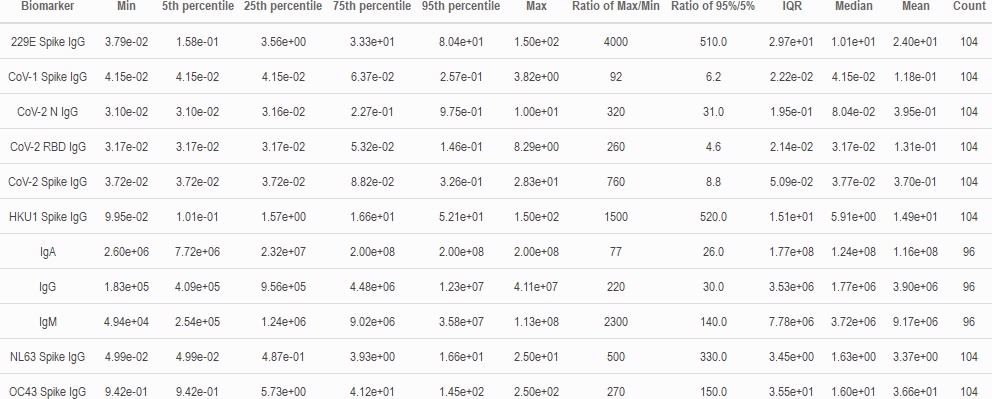


Supplementary Table 3. Immunoglobulin concentrations in saliva self-collected by donors without confirmed COVID-19 diagnosis, household exposure, or recent symptoms. These measurements were used to establish the upper-limit of non-specific reactivity. Concentrations for total immunoglobulins (IgA, IgG, and IgM) are reported in pg/mL. All other concentrations are AU/mL.


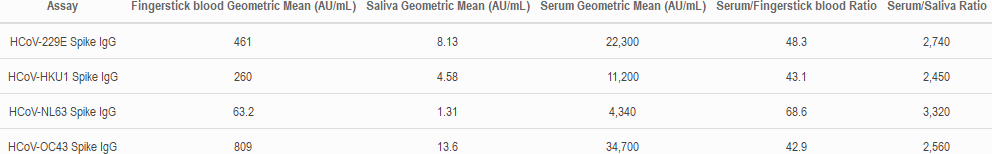


Supplementary Table 4. Antibody concentrations for endemic coronaviruses in finger-stick blood, saliva, and serum. Geometric means were computed for IgG concentrations measured in finger-stick blood and saliva for four endemic coronaviruses, and compared to previously measured values for serum. Ratios of these values were computed to determine the relative amounts of antibodies in serum versus finger-stick blood and saliva. Averages of these ratios were 50.1x for finger-stick blood and 2,800x for saliva.


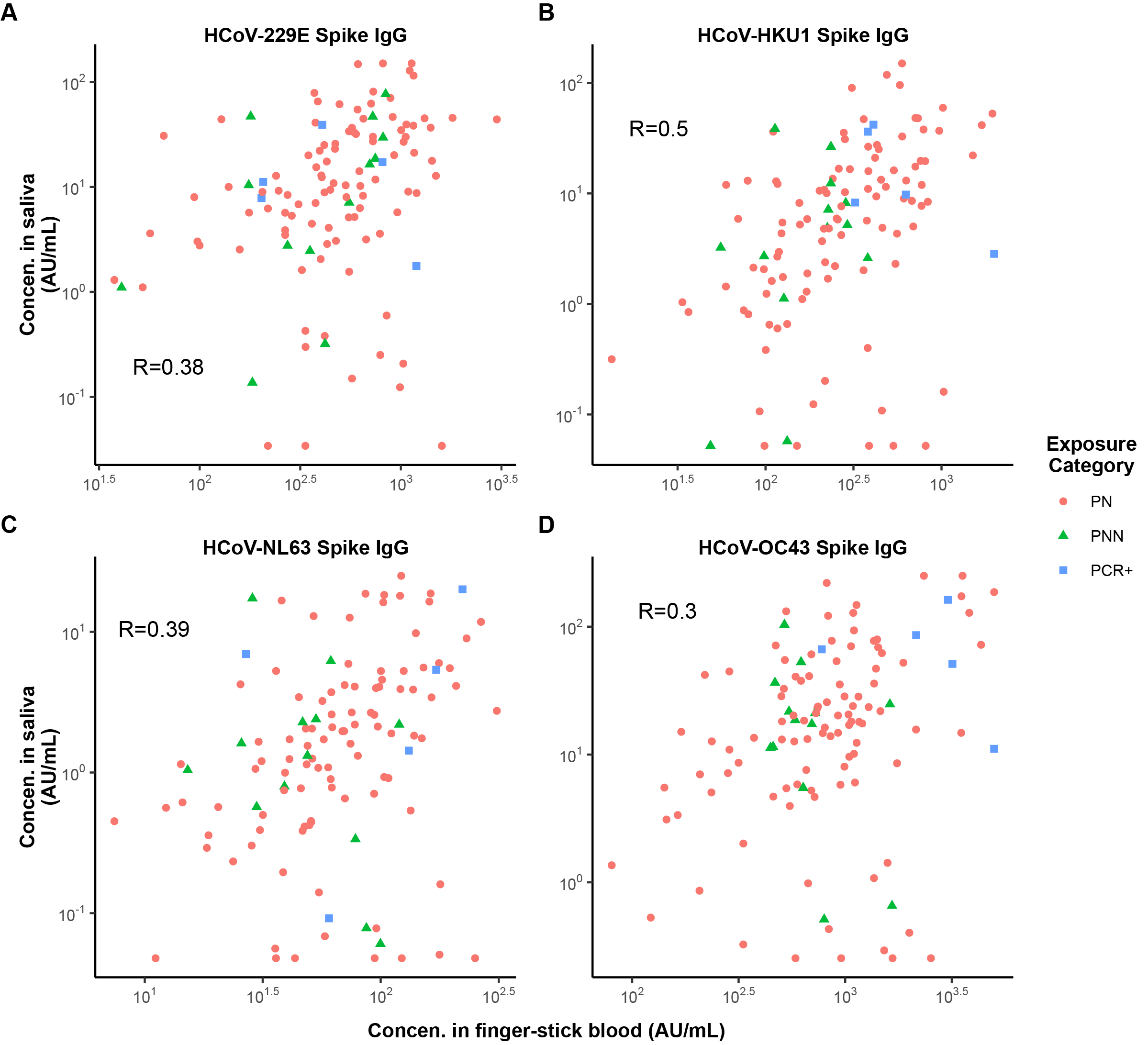


Supplementary Figure 7. Correlation in reactivity to CoV-2 antigens measured in self-collected saliva versus finger-stick blood. Levels of IgG antibodies were measured in matched saliva and finger-stick blood provided by the same donors. Dotted lines indicate the selected classification thresholds. For each figure, the lower left quadrant contains samples that are within the range of non-specific reactivity for both saliva and finger-stick blood. The upper right quadrant are samples with high reactivity for both saliva and finger-stick blood.
